# How plastic are upper thermal limits? A comparative study in tsetse (family: Glossinidae) and wider Diptera

**DOI:** 10.1016/j.jtherbio.2023.103745

**Published:** 2023-10-30

**Authors:** Hester Weaving, John S. Terblanche, Sinead English

**Affiliations:** a School of Biological Sciences, University of Bristol, Bristol, UK; b Department of Conservation Ecology & Entomology, Stellenbosch University, Stellenbosch, South Africa

**Keywords:** Heat tolerance, Acclimation, Critical thermal maximum, Body size, Temperature stress

## Abstract

Critical thermal maximum (CT_max_) describes the upper thermal tolerance of an animal where biological functions start to fail. A period of acclimation can enhance CT_max_ through plasticity, potentially buffering animals from extreme temperatures caused by climate change. Basal and acclimated CT_max_ vary within and between species and may be explained by traits related to thermal physiology, such as body size and sex. Differences in CT_max_ have not been established among species of tsetse fly (*Glossina* spp.), vectors of animal and human African trypanosomiasis. Here, we investigated basal CT_max_ and its plasticity for five tsetse species following adult acclimation at constant 25 or 30 °C for five days. We then set our findings in context using a meta-analysis on 33 species of Diptera. We find that, of the five tsetse species considered, only *Glossina palpalis gambiensis* and *Glossina brevipalpis* exhibited plasticity of CT_max_, with an increase of 0.12 °C and 0.10 °C per 1 °C acclimation respectively. Within some species, higher basal CT_max_ values were associated with larger body size and being female, while variation in plasticity (i.e., response to the acclimation temperature) could not be explained by sex or size. Our broader meta-analysis across Diptera revealed overall CT_max_ plasticity of 0.06 °C per 1 °C acclimation, versus a similar 0.05 °C mean increase in tsetse. In contrast, there was greater CT_max_ plasticity in males compared to females in Diptera. Our study highlights that CT_max_ and its plasticity varies even among closely related species. Broader patterns across groups are not always reflected at a finer resolution; we thus emphasise the need for detailed experimental studies across a wide range of insect species to capture their capacity to cope with rapidly warming temperatures.

## 1 Introduction

Thermal tolerance can be defined by upper and lower critical thermal limits, CT_max_ and CT_min_, or thermal tolerance breadth (Angilletta, 2009). CT_max_ is one of the most important predictors of species’ distributions (Kellermann et al., 2012; Overgaard et al., 2014), so can be used as an indicator of vulnerability to climate change. A period of acclimation can enhance CT_max_ through plastic responses (Allen et al., 2012; Belliard et al., 2019), which could act as a mechanism to buffer animals during periods of extreme heat and increased temperature variability, two phenomenon becoming more prevalent due to climate change (Christidis et al., 2015; Meehl and Tebaldi, 2004; Perkins et al., 2012). CT_max_ can be assessed by a dynamic assay where temperature is ramped until a performance endpoint − for example, no response to prodding, the onset of muscle spasms, or the inability to cling to a surface (Terblanche and Chown, 2006). Thermal plasticity can be estimated as the difference between the CT_max_ of a population held under benign (optimal) conditions, compared to a population which was acclimated in an elevated, but nonlethal, temperature before the assay. Thermal reaction norms are the relationship between these two measures, with plasticity equivalent to the slope. Acclimation response ratio (ARR) is the change in critical thermal limit per degree change in acclimation temperature, which describes this slope (Angilletta, 2009).

Comparative analyses across ectothermic species have shown that thermal tolerance varies predictably across seasonal (Clusella-Trullas and Chown, 2014; Oliveira et al., 2021), latitudinal (Addo-Bediako et al., 2000; Clusella-Trullas and Chown, 2014), and elevational clines (García-Robledo et al., 2016). There is less consistent evidence about the relationship between body size and thermal tolerance. Studies suggest that a wide variety of animals are decreasing in size in response to rising temperatures (Gardner et al., 2011; Sheridan and Bickford, 2011), and past extinction events due to warming have selected for smaller bodied marine and terrestrial animals (Sheridan and Bickford, 2011; Smith et al., 2009). Paradoxically, larger animals may have higher basal CT_max_ due to their slower metabolic rate relative to body size and the ability to store more resources (Brown et al., 2004; Kingsolver and Huey, 2008). Additionally, surface area to volume ratio scales negatively with size, so large animals may suffer less from water loss (Addo-Bediako et al., 2000; Bergmann, 1847; Chown et al., 2011). The association between large body size and high CT_max_ has been found both within and between species in ants (Baudier and O’Donnell, 2018), frogs (von May et al., 2019), and fish (Zhang and Kieffer, 2014). However, lower values for CT_max_ have been found with increasing body size for fish (Recsetar et al., 2012), crustaceans (Verberk et al., 2018), and other marine animals (Peck et al., 2009). This relationship may be exclusive to aquatic animals because a small surface area-to-volume ratio limits large bodied animals to extract oxygen from water at temperature extremes (Chapelle and Peck, 1999; Pörtner, 2010). An analysis of over 328 species (including marine and terrestrial ectotherms) found a more complex relationship where large-bodied animals are less tolerant than small animals to acute heat, but were more heat tolerant during long exposure times (Peralta-Maraver and Rezende, 2021).

Broad-scale comparative analyses of upper and low thermal tolerance plasticity have found mixed support for the hypothesis that selection promotes plasticity in variable thermal environments, such as with increasing latitude (Donelson et al., 2018). These studies often show opposing trends, or fail to explain much variation in ARR (Gunderson and Stillman, 2015; Seebacher et al., 2015; Weaving et al., 2022). Once more, few studies have investigated the relationship between thermal tolerance plasticity and body size (but see Rohr et al., 2018) despite its potential relationship to thermal experience (Pincebourde et al., 2021). Larger animals have greater thermal inertia so perhaps change body temperature more slowly and therefore may be slower to acclimate (Rohr et al., 2018). Additionally, lifespan tends to increase with body size, and longer lifespans may be subject to a greater thermal range e.g., over annual rather than seasonal scales (Rohr et al., 2018). A meta-analysis examining over 500 species of ectotherms by Rohr et al. (2018) found that ectothermic animals with larger body sizes had greater plastic responses at longer acclimation times, and at slower assay ramping rates.

Upper thermal tolerance and its plasticity can also vary according to an individual’s sex, due to sexual dimorphism and behavioural differences. Males tend to express more risk-taking behaviours and inhabit larger ranges, which could expose them to greater temperature variability, promoting greater plasticity (Stillwell et al., 2010; Tarka et al., 2018). However, female ectotherms tend to be larger, perhaps acting in opposition to this trend due to, for example, greater resources and more efficient resource use, as outlined above (Bulté and Blouin-Demers, 2010). In a meta-analysis of 44 ectothermic species, Pottier et al. (2021) found that females were more plastic than males, but only in field-caught individuals. However, in a meta-analysis specific to insects (102 species), Weaving et al. (2022) found no sex differences in thermal tolerance plasticity.

**Fig. 1:**
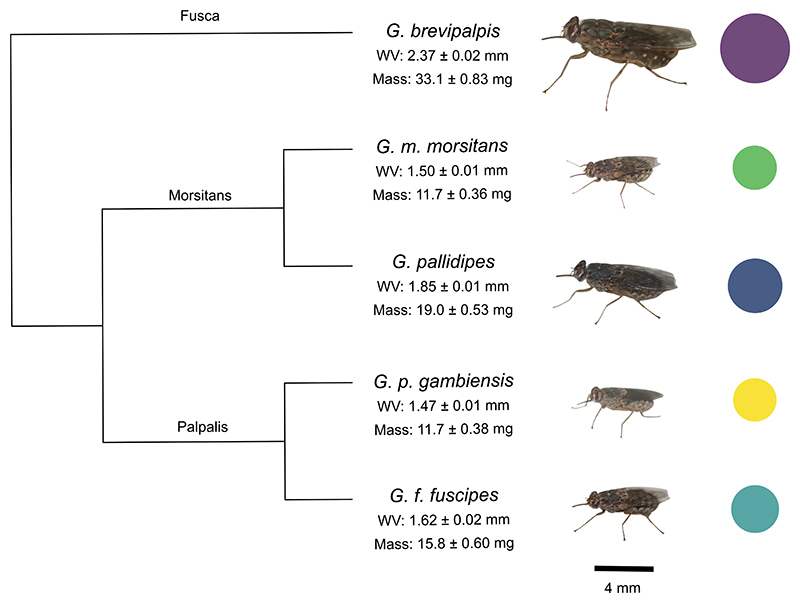
Photographs of each tsetse species (*Glossina* spp.) used in the experiment, to scale by wing vein (WV; mm). Dry mass is given in mg. Pictured are adult females of each species. These species represent the full range of subgenera: Fusca (*G. brevipalpis*), Morsitans (*G. m. morsitans, G. pallidipes*), and Palpalis (*G. f. fuscipes, G. p. gambiensis*). Phylogeny was constructed using Open Tree of Life and R packages ‘rotl’ and ‘ape’ in R and shows the five species measured in this experiment of the total 31 species and subspecies. Circles represent relative body size by wing vein and colours used in Figures throughout.

Forecasting responses to climate change is particularly important for vectors of disease, as changes to distribution may result in altered disease transmission (Hay et al., 2004; Rogers and Randolph, 1993; Simarro et al., 2012). Many disease vectors, such as mosquitoes, have short generation times and high population growth rates which promote evolutionary adaptation (Burger and Lynch, 1995; Couper et al., 2021). In contrast, tsetse flies (*Glossina* spp.), vectors of trypanosome parasites, are slow to reproduce and population persistence is highly sensitive to temperature (Buxton, 1955; Hargrove, 2004). Therefore, in a warming world, tsetse may need to rely on within-lifetime plastic thermal tolerance, rather than across-generation changes. However, thermal tolerance and its plasticity have been quantified in *Glossina pallidipes* by Terblanche and Chown (2006), who found no evidence of adult or developmental plasticity in CT_max_. It is unknown if patterns observed in *G. pallidipes* reflect the entire genus indicating constraints to CT_max_ plasticity or if there is systematic variation across the genera. There are 31 species and subspecies of *Glossina*, which are split into 3 subgenera: Morsitans, Palpalis and Fusca, with differing habitat preferences (Leak, 1998). Morsitans flies largely inhabit savanna and woodland, Palpalis inhabit environments with rivers and lakes, and Fusca are generally found in the moist forests of West Africa, although *Glossina brevipalpis* occurs discontinuously throughout the tsetse belt. The various tsetse species also cover a range of body sizes, for example the body mass of *G. brevipalpis*, one of the largest species, is five times as great as one of the smallest species, *Glossina austeni* (Leak, 1998), making them an interesting group to explore thermal tolerance variation (Fig. 1).

Here, we measure the critical thermal maximum (CT_max_) and its plasticity across five tsetse species (*G. brevipalpis, G. pallidipes, Glossina fuscipes fuscipes, Glossina morsitans morsitans, Glossina palpalis gambiensis*). The largest species is *G. brevipalpis*, which is three times greater in body mass than the smallest species measured, *G. p. gambiensis* (Fig. 1). These species cover all three subgenera, originating from a range of locations (Supplementary Table 1). We investigate within and between species differences in basal and acclimated CT_max_ and ask how these relate to body size and sex. We expect larger body sizes to give rise to higher basal CT_max_, and greater plasticity to be associated with large body size. We expect no differences in plasticity between sexes due to competing selection pressures. We then set our results in the context of a meta-analysis on 33 species of Diptera to confirm if patterns across the five species in this unique family reflect more broad findings across the order.

## 2 Materials and methods

### 2.1 Pupal development and adult emergence

Approximately 300 early-stage pupae (within around one week of deposition) of five tsetse species (*G. brevipalpis, G. m. morsitans, G. pallidipes, G. f. fuscipes, G. p. gambiensis*; Fig. 1) were ordered from the International Atomic Energy Agency (IAEA), Vienna, between October 2022 and February 2023. IAEA colony conditions are 24−25 °C and 75−80 % Relative Humidity (RH) for adult tsetse and 23−24 °C and 75−80 % RH for pupae (Opiyo et al., 2006). By using laboratory-reared individuals, we eliminate the possibility that differences in plasticity come from varying thermal history e.g. developmental plasticity from different rearing environments (van Heerwaarden and Kellermann, 2020). Once delivered, pupae were kept at 25 °C and 80 % RH in a climate-controlled room, monitored by an iButton at a sampling frequency of 30 min. Adults and pupae were kept in the same climate-controlled room, so the same conditions were used for both stages. The light:dark cycle was 12:12, 9am − 9pm using dimmed lighting. Pupae were housed in emergence cages (approx. 150 pupae per cage) and covered with sterilized sand.

Upon emergence, adults were separated from pupae and transferred to a chest fridge to be sorted into single sex cages (maximum 25 flies per cage). The fridge was maintained between 2 and 6 °C using a RS Pro Dual Datalogger with T type thermocouples, and flies were held at this temperature for no longer than 5 min. Cages were made from modified plastic piping (16 cm diameter x 8 cm depth) with mesh fabric (2.5 mm holes) on the top and bottom, with a circular opening bunged with a cork. Females emerge before males so approximately eight cages of females were collected on days one and two, and eight cages of males were collected on days three and four, although actual numbers varied per species. Adult flies were kept in the climate-controlled room on racks at the above-mentioned conditions.

### 2.2 Feeding

Defibrinated horse blood (TCS Biosciences, Buckingham, UK) was ordered in 500 ml quantities and decanted mechanically (Rota-filler 3000) using a 50 ml serological pipette (Sarstedt) into 25 ml universals (Sterilin) under a laminar flow hood. Blood was stored in the fridge at ∼4 °C for no more than 3 weeks. Flies were fed the day after being sorted and then three times weekly on Monday, Wednesday, and Friday at approximately 9:00 a.m. 200 μl of ATP was added to each 25 ml vial of blood using a 100−1000 μl pipette (Eppendorf) as a feeding stimulant. ATP was made by diluting 5.51 g of adenosine 5′-triphosphate disodium per 100 ml of Reverse Osmosis water and mixed using a Corning Stirrer PC-353 with magnetic flea.

Blood was poured on to metal trays (25 ml per 47 cm × 40 cm tray), covered with a silicon membrane, and heated to 36 °C using heated mats (Flexible heated hoses, Birmingham, UK). A thermocouple was used to monitor temperature. After feeding, trays and membranes were rinsed with cold water, scrubbed, and sterilized at 110 °C overnight in an oven (Gallenhamp, Hotbox Oven).

### 2.3 Acclimation

Flies emerged on day zero, were fed on day one and then were transferred to their acclimation treatments at 11:00 a.m. Treatments were constant 25 °C as the control (i.e., basal) temperature and 30 °C for the acclimation temperature for a period of five days. An acclimation treatment of 30 °C was used because it is near to the constant upper temperature at which tsetse can survive, being around 32 °C for *G. p. gambiensis* and *G. m. morsitans*, but depends on species (Are and Hargrove, 2020; Pagabeleguem et al., 2016). Temperatures within and above this range are regularly experienced in the field: for example, at Rekomitjie Research Station, Zimbabwe, maximum air temperatures can reach 42 °C (see temperature data in Supplementary data for Lord et al. (2018)). Half of the flies were transferred to 30 °C, 80% RH in an incubator (Snijder Micro Clima-Series) with 12:12 light:dark conditions, and half remained at 25 °C in the climate-controlled room. Flies housed in the incubator were kept in a large box drilled with holes for ventilation. The box was covered with blue roll to create similar dim lighting conditions as the climate-controlled room. Flies were only removed from the acclimation treatment to feed, three times weekly, as described above. Feeding occurred within the climate-controlled room at 25 °C. For all species, the actual mean temperature (°C) and relative humidity (RH %) experienced in the 25 °C treatment was 24.9 ± 0.2 and 78.2 ± 0.8, and in the 30 °C acclimation treatment was 30.6 ± 0.7 and 79.9 ± 4.3, respectively. Mean temperature and humidity data for individual species are given in Supplementary Table 1.

### 2.4 CT_max_ assay

CT_max_ assays were undertaken using two programmable Grant LTC4 refrigerated circulating liquid baths with TX150 heating circulators, attached to a set of Perspex organ pipes with rubber tubing (Supplementary Fig. 1), and filled with water. The temperature program was set using Grant Labwise software (Version 2.1.2, Grant Instruments, Cambridge, UK) and consisted of 10 min acclimation at 25 °C followed by a ramping treatment at a rate of +0.1 °C/min. Ramping rates within this range have been used for tsetse in previous studies and this rate can be considered ecologically relevant from microsite temperature profiles in the field (Terblanche and Chown, 2006). Two thermocouples (Type T) monitored temperature in one empty tube per water bath during the experiment. Fly temperature was considered the same as tube temperature due to the small body size of tsetse, as previously determined (Terblanche and Chown, 2006). Four runs were completed for each species (n = ∼80 flies per species across four runs), half of individuals were male and half female, with an equal number from each acclimation treatment.

Flies were fed on the last day of the acclimation treatment (day five) so that all individuals had taken a bloodmeal on the previous day. Assays began at approximately 11 a.m. on day six, although start time varied depending on run (9:30−13:30). This meant that flies were approximately one week old on the day of the thermal assay. Flies were knocked down using 100 % Sevoflurane inhalation anaesthetic (SevoFlo, Zoetis, Belgium). Sevoflurane was chosen as an anaesthetic as it has minimal effects on survival and reproduction in comparison to cold anaesthetic in *Drosophila* (MacMillan et al., 2017). In a separate experiment, to ensure that sevoflurane did not negatively affect tsetse, cages of sevoflurane-treated and non-treated flies were assessed for mortality after one week. We used a glm with quasibinomial family and “logit” link to analyse these data. We found no significant difference between the sevoflurane-treated and non-treated groups (mean difference ± SE = - 0.15 ± 0.43, z value = −0.34, p = 0.74). Proportion mortality data can be found in Supplementary Table 2. Sevoflurane (350 μl) was applied to cotton wool for one cage of approximately 25 flies in an enclosed plastic container (20 × 25 × 10 cm) for 10 min. Ten flies were randomly selected per treatment and rapidly transferred into the organ pipes using stork bill forceps and bunged with cotton wool and a cork. Flies allocated to each treatment were placed in the pipes alternately. Tsetse were allowed to recover from anaesthetic knock down, so that all flies were standing upright before the temperature program began. In all cases, standing occurred less than 10 min post anaesthetic. Occasionally (n = 2/397) dead flies were selected from cages by mistake, and these individuals were excluded from the assay.

One researcher (HW) took all CT_max_ measurements to avoid observer bias. Programs on the two water baths began in 20−30-min tandem so that only 10 flies were assessed at once. CT_max_ was defined as the temperature at which the fly was knocked down or lost locomotor ability and stopped responding to a stimulus (disturbance by rocking the organ pipes). After the temperature rose to 40 °C (known to be near to tsetse CT_max_ from pilot trials and previous research on *G. pallidipes* under these experimental conditions), flies were checked every 30 s for movement. Once all flies were knocked down, they were removed from the pipes and placed into 50 ml conical falcon tubes to assay subsequent mortality, each drilled with a hole in the lid for ventilation.

Mortality was determined by shaking the tube 24 h after the assay. Flies were considered dead if they could not right themselves. Mortality was expected to be close to 100% as CT_max_ is usually near or the same as lethal temperature in insects (Vorhees and Bradley, 2012). Flies were frozen at -18 °C for 24 h and then dried overnight in an oven at 70 °C. Dry mass was taken on a Ohaus Explorer EX124 balance (accurate to 1/1000 mg). The left wing was removed and photographed using a Leica EZ4W dissecting camera microscope at 35x magnification and LAS EZ (Version 3.4.0). ImageJ (Version 1.53) was used to take the size of the hatchet cell wing vein length, known to indicate fly size (Jackson, 1946), and this was calibrated using a graticule with 0.1 mm divisions.

### 2.5 Statistics

All analyses were completed in R (version 4.2.3; R Core Team, 2021). Raw data can be found in the Supplementary files. We used mixed-effect linear models using the lme4 package (version 1.1−31; Bates et al., 2015) with experimental run as a random effect. We started from a maximal model and each term was excluded and removed if it did not significantly improve model fit (Bradburn et al., 2003). The difference between models was tested using analysis of variance (ANOVA). Where interactions were significant, we split the data into separate models so that interactions could be investigated fully. These models did not contain experimental run as a random effect as there were only four runs per treatment. Post-hoc pairwise comparisons were calculated using the ‘lsmeans’ package (Version 2.30; Lenth, 2016) and P-values were adjusted using the false discovery rate method (FDR).

Linear mixed-effects models were validated by plotting standardised residuals against fitted values to check for heteroscedasticity. Normality of residuals were validated using a Q-Q plot and Shapiro-Wilk test. Influential data points were identified using Cook’s distance. One *G. m. morsitans* outlier was removed from the data set due to being highly influential. Once this outlier was removed all model residuals were normal. Wing vein length and dry body mass were highly positively correlated (t_1, 393_ = 39.8, p < 0.001, R^2^ = 0.80). Wing vein was used preferentially in models as mass varies due to the quantity of the blood meal last taken. There was also a relationship between size and sex, with male flies smaller than female flies (mean difference ± SE = −0.22 ± 0.03, t_1, 393_ = 40.3, p < 0.001, R^2^ = 0.093), so all regressions were rerun, replacing sex with size to eliminate nonindependence issues.

Figures were created using packages ggplot2 (Version 3.4.2; Wickham, 2016) and viridis for the colour palette (Version 0.6.2; Garnier et al., 2023). Fig. 4 was created using the orchaRd package (Version 2.0; Nakagawa et al., 2021).

#### 2.5.1 Basal heat tolerance

First, we assessed variation in basal CT_max_ according to species, sex, and body size. Data from individuals kept at 25 °C were considered. We modelled CT_max_ as a function of species and size (using wing vein), and the interaction between these variables. We considered species differences using species as a fixed effect term rather than conducting a phylogenetic analysis due to relatively low statistical power (due to measuring 5 of the total 31 tsetse species and subspecies for logistical reasons). We then repeated the analyses with sex rather than body size, as explained previously.

#### 2.5.2 Acclimation responses

To determine within and between species differences in adult plasticity of CT_max_, we used data from individuals across both acclimation treatments. Treatment (acclimation at 25 °C or 30 °C), body size, species, and interactions between these variables (up to three-way) were considered as fixed factors. A significant interaction between treatment and size, or treatment and species, would indicate size- or species-dependent plasticity in CT_max_. Regressions represent the reaction norm for each species, with the slope of the line equivalent to the degree of plasticity. Here we have assumed that the reaction norm between 25 °C and 30 °C groups is linear, although we acknowledge this is not always the case (van Heerwaarden and Kellermann, 2020). Future studies could explore a wider range of acclimation temperatures to test this assumption.

### 2.6 Acclimation responses within Diptera

We calculated the acclimation response ratio (ARR) using ARR = $\frac{CTL_{[{\text{T}}_{2]}}-CTL_{\left[{\text{T}}_{1}\right]}}{{\text{T}}_{2}-{\text{T}}_{1}}$ for each tsetse species (Cossins and Bowler, 1987). CTL_[T1]_ and CTL_[T2]_ are the CT_max_ at 25 (T_1_) and 30 °C (T_2_). ARR represents the change in CT_max_ per 1 °C temperature increase. A comparison was made to other Dipterans and within the *Glossina* genus, using data from Weaving et al. (2022). An additional literature search was completed to find any new or missing literature for tsetse and mosquitoes, as similar blood feeding vectors of disease. We searched Web of Science using the following terms: (mosquito*) AND (thermal OR heat OR temperature) AND (CTmax* OR critical thermal max*) AND (plastic* OR (phenotyp* plastic*) OR acclim* OR stress OR tolerance) NOT (mosquitofish*). This resulted in seven additional articles, one of which had useable data. For Google Scholar and Scopus, the following search terms were used: *“mosquito” ctmax plasticity*. These differed due to the first set of Boolean terms not being accepted in these search engines. Scopus had nine results and one had appropriate data for extraction. Abstracts on the first three pages of Google Scholar were examined, two of which had useable data. Hits after the first three pages became irrelevant so were not examined for further articles. The same search was performed for tsetse, replacing “mosquito” with “tsetse” in the search terms, and removing “*NOT “mosquitofish”*“. No new articles were found for tsetse on the three search engines. Digitizer was used to extract data from graphs (Rohatgi, 2010). References for studies used in the meta-analysis can in the Supplementary data. For further detail on methodology, see Weaving et al. (2022).

The R package ‘metafor’ (Version 3.0−2; Viechtbauer, 2010) was used to perform a multi-level, random effects model comparing ARR within Diptera. The model was run with random effect structure as determined in Weaving et al., (2022), i.e. study ID, phylogeny, species ID, and effect size ID. The phylogenetic tree was constructed in the Open Tree of Life and R packages ‘rotl’ (Michonneau et al., 2016; Version 3.0.11) and ‘ape’ (Paradis and Schliep, 2019; Version 5.5) and a phylogenetic correlation matrix was constructed based on hypothetical relatedness of species which was also included in the model. Family group, sex and body mass were used as moderators to explain variation in ARR. Dipteran families included: Glossinidae (tsetse flies), Culicidae (mosquitoes), Drosophilidae and Tephritidae (fruit flies), and Ceratopogonidae (biting midges). Publication bias was assessed by Egger’s regression test (Nakagawa et al., 2022; Weaving et al., 2022).

## 3 Results

### 3.1 Basal heat tolerance

Overall, basal CT_max_ differed among species by a maximum of 1.8 °C (χ^2^ = 11.19, df = 4, p = 0.02), for which mean values are presented in Supplementary Table 3. Basal CT_max_ was ordered from highest to lowest by species as follows: *G. f. fuscipes, G. pallidipes*, equally *G. m. morsitans* and *G. p. gambiensis*, and finally *G. brevipalpis*.

We expected higher basal CT_max_ in larger flies due to greater resources and reduced metabolic rate per unit mass. Indeed, we found that larger tsetse had greater basal CT_max_ (χ^2^ = 4.31, df = 1, p = 0.04), but a significant interaction between species and body size improved model fit, indicating within species relationships (χ^2^ = 9.03, df = 4, p = 0.06). Single species models illustrated that basal CT_max_ increased with size for *G. m. morsitans* (F = 2.77, df = 1, p < 0.001), *G. pallidipes* (F = 8.40, df = 1, p = 0.006), and there was a non-significant trend for *G. brevipalpis* (F = 3.35, df = 1, p = 0.08). There was no significant relationship for *G. f. fuscipes* (F = 0.76, df = 1, p = 0.39) or *G. p. gambiensis* (F = 1.74, df = 1, p = 0.20; Supplementary Fig. 2).

Overall, males had lower basal CT_max_ than females (χ^2^ = 7.50, df = 1, p = 0.006). However, again, this relationship was dependent on species (χ^2^= 1.61, df = 4, p = 0.003). Single-species models showed that females had greater basal CT_max_ than males for *G. m. morsitans* (F = 26.0, df = 1, p < 0.001), *G. pallidipes* (F = 6.69, df = 1, p = 0.01), *G. brevipalpis* (F = 4.55, df = 1, p = 0.04). *Glossina fuscipes fuscipes* females also had greater CT_max_, but the trend was non-significant (F = 3.06, df = 1, p = 0.09). These results are consistent with those found for CT_max_ and body size, as female tsetse tend to be larger. There was no relationship between sex and CT_max_ for *G. p. gambiensis* (F = 0.42, df = 1, p = 0.52).

### 3.2 Acclimation responses

**Fig. 2:**
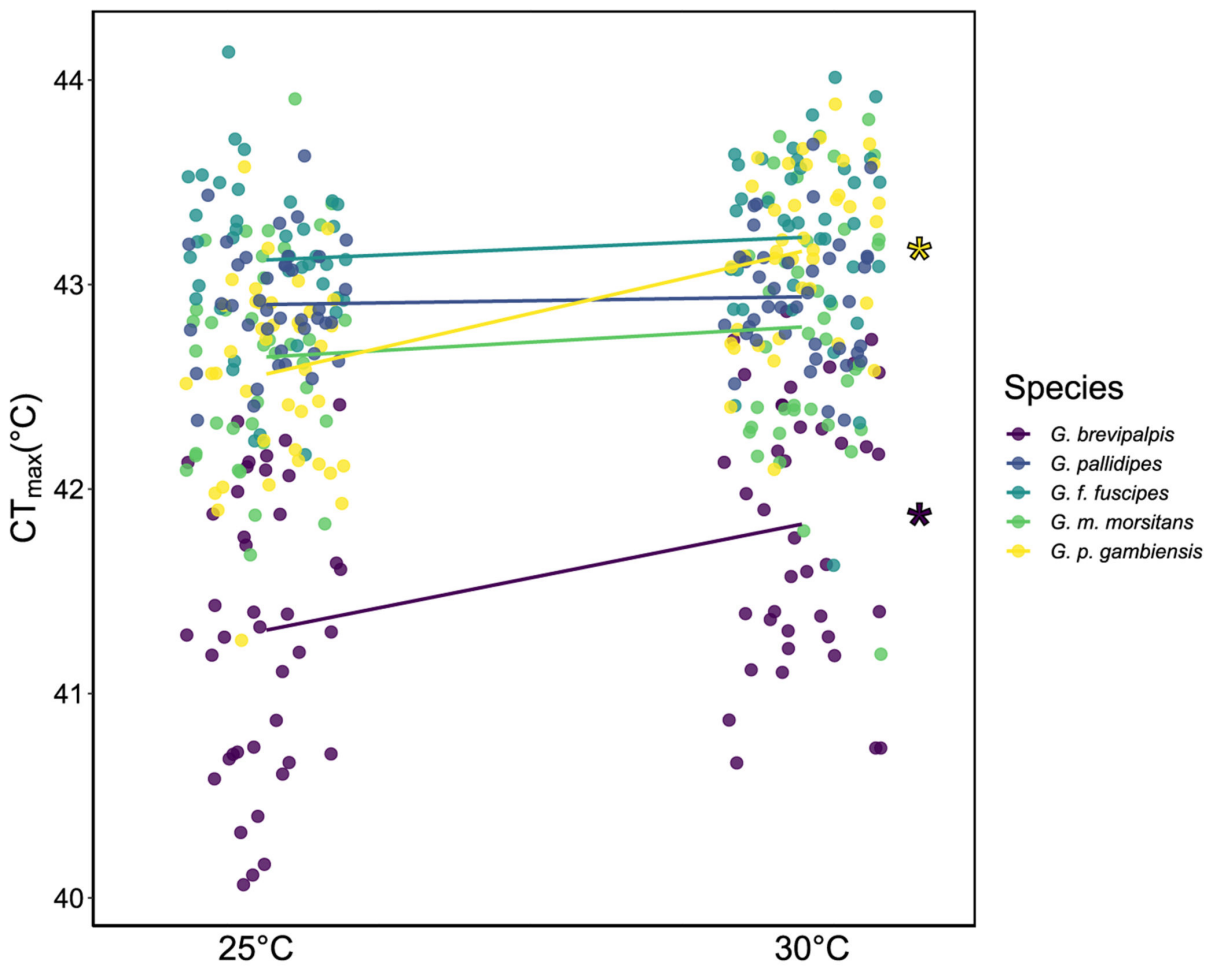
Critical Thermal Maximum (CT_max_) after acclimation at basal (25 °C) and elevated (30 °C) temperature for five days. *Glossina* spp. are distinguished by different colours and are presented in the legend in size order by wing vein. Lines represent the reaction norm of each species with the slope equivalent to plasticity. Significant differences between the CT_max_ of the two acclimation temperatures is indicated by an asterisk, which is seen in *G. brevipalpis* and *G. p. gambiensis*. N ∼40 per treatment/species.

We investigated how five-day acclimation at 30 °C affected CT_max._ Overall, CT_max_ increased by 0.06 °C per 1 °C rise in acclimation temperature (χ^2^ = 26.7, df = 1, p < 0.001), but there was variation in plasticity among species (Supplementary Tables 3 and 4; χ^2^ = 24.5, df = 4, p < 0.001). Single-species models showed that acclimation increased CT_max_ for *G. p. gambiensis* (F = 36.8, df = 1, p < 0.001) and *G. brevipalpis* (F = 13.6, df = 1, p < 0.001) by 0.12 °C and 0.10 °C per 1 °C acclimation respectively (Fig. 2). There was no change in CT_max_ for *G. m. morsitans* (F = 2.68, df = 1, p = 0.11), *G. pallidipes* (F = 0.37, df = 1, p = 0.55), and *G. f. fuscipes* (Fig. 2; F = 1.27, df = 1, p = 0.26) of 0.04, 0.01, and 0.02 per 1 °C acclimation respectively. Post-hoc analysis examining species-level differences between reaction norm slopes found that *G. p. gambiensis* was the most plastic species, with a significantly steeper reaction norm than *G. f. fuscipes, G. m. morsitans* and *G. pallidipes* (Table 1). *Glossina brevipalpis* was the second most plastic, having a significantly steeper reaction norm than *G. f. fuscipes* and *G. pallidipes* (Table 1).

**Fig. 3:**
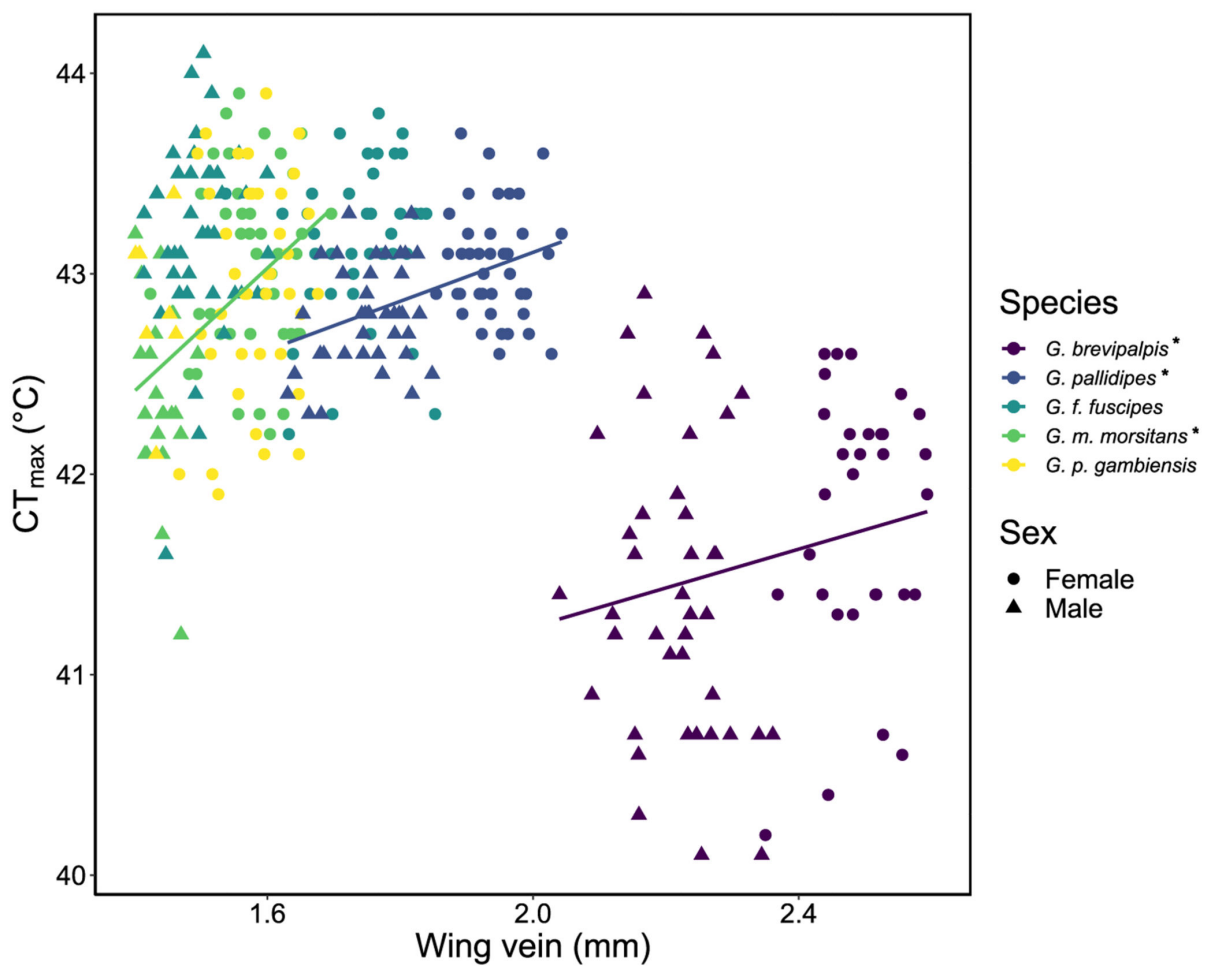
Relationship between CT_max_ and wing vein length (mm). *Glossina* spp. are distinguished by different colours. Lines represent linear regressions for the three species groups where these were significant, i.e., *G. brevipalpis, G. pallidipes* and *G. m. morsitans*. The same three species had females with significantly greater CT_max_ than males, which is denoted by an asterisk. Circles resemble female flies and triangles resemble males. Species are given in size order in the legend from largest to smallest by wing vein. CT_max_ is represented for individuals acclimated at both 25 °C and 30 °C. N ∼40 per treatment/sex/species.

We predicted that larger flies would be more plastic, however we found no relationship between plasticity and body size (χ^2^ = 0.006, df = 1, p = 0.94), therefore we present the relationship between size and both basal and acclimated CT_max_ data in Fig. 3. Acclimated and basal CT_max_ increased with body size (χ^2^ = 4.50, df = 1, p = 0.03), but this relationship depended on the species tested (χ^2^ = 12.3, df = 4, p = 0.01). Single species models revealed that larger flies had greater CT_max_ for *G. pallidipes* (F = 19.0, df = 1, p < 0.001), *G. m. morsitans* (F = 32.0, df = 1, p < 0.001), and *G. brevipalpis* (F = 3.91, df = 1, p = 0.05). However, there was no relationship for *G. f. fuscipes* (F = 0.06, df = 1, p = 0.80) and *G. p. gambiensis* (F = 0.24, df = 1, p = 0.26), in accordance with models based on only basal CT_max_. For comparison, we also give these relationships with dry body mass in Supplementary Fig. 3.

We predicted that males would be more plastic than females, but we found no difference in plasticity between sexes (χ^2^ = 0.54, df = 4, p = 0.46). However, there was a significant interaction between species and sex (χ^2^ = 24.1, df = 4, p < 0.001), and species and treatment (χ^2^ = 24.7, df = 4, p < 0.001). When these data were split into single species models, we found similar patterns as between body size and CT_max_ − females had greater CT_max_ for *G. pallidipes, G. m. morsitans* and *G. brevipalpis* (Fig. 3; Supplementary Table 4).

### 3.3 Acclimation responses within Diptera

**Fig. 4:**
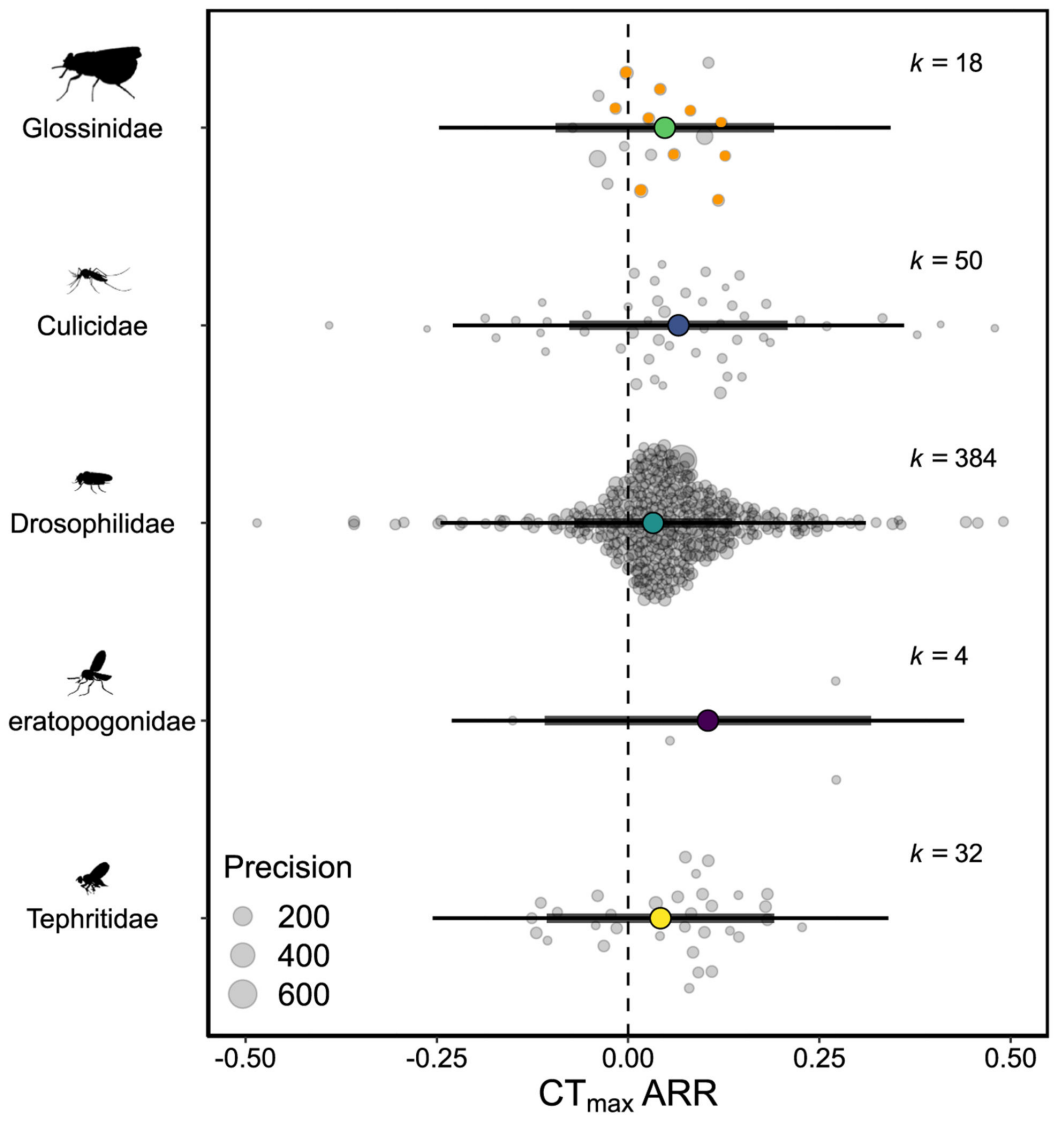
Acclimation Response Ratio (ARR) of Critical Thermal Maximum (CT_max_ in °C) across five Dipteran families. Effect sizes from the current study are highlighted in orange, males and females are displayed separately giving n = 10. A positive ARR indicates an adaptive plastic response, whereby heat acclimation increases CT_max_. 95% confidence intervals (95% CIs) are depicted in heavy black lines, prediction intervals in thin black lines. The size of each data point is proportional to 1/SE (Standard Error), indicating the precision of the study. k = number of effect sizes per group. One effect size from Drosophilidae was excluded from the Figure so a smaller axis could be presented. Icons are roughly scaled by the size of the family group, icon credit: phylopics.

A total of 488 effect sizes (from 25 studies, 33 species) were calculated to examine the effect of acclimation on CT_max_ in Diptera. Drosophilidae were by far the most represented family (k = 384), followed by Culicidae (mosquitoes; k = 50), Tephritidae (fruit flies; k = 32), Glossinidae (tsetse flies; k = 18), and Ceratopogonidae (biting midges; k = 4). Overall, we found for every 1 °C rise in acclimation temperature, CT_max_ increased by 0.048 °C (Table 2; 95% CI = 0.024, 0.072). Therefore, overall plasticity of *Glossina* species (0.06 °C) is similar to Diptera (0.05 °C). We assessed whether variation in plasticity of CT_max_ in Diptera was explained by moderators (sex, body mass, family) using a series of univariate models. ARRs are stated as mean differences between groups (with the direction of comparison stated in subscript) or as a meta-regression for body mass. We found that males were slightly more plastic than females (ARR _male-female_ = 0.026; 95% CI = 0.003, 0.048), but found no differences in plasticity between families or a relationship with dry body mass (Table 2; Fig. 4). There was no significant publication bias (βARR = 0.019; 95% CI = −0.45, 0.49; Supplementary Fig. 4).

## 4 Discussion

We found that acclimation to an elevated temperature (30 °C versus 25 °C) across five days in early adulthood enhanced CT_max_ in two of the five tsetse species measured, but differences in plasticity were not associated with body size or sex. Within some tsetse species, higher basal CT_max_ values were associated with larger body size and being female, but these differences were not found between species i.e. the largest species *G. brevipalpis* actually had the lowest basal tolerance. Our broader meta-analysis revealed similar mean acclimation responses between tsetse and Diptera, of 0.05 and 0.06 per 1 °C acclimation respectively. In contrast to tsetse, we found greater CT_max_ plasticity of male Dipterans compared to females.

The two of the five species that responded to acclimation were *G. p. gambiensis* and *G. brevipalpis*, with a 0.12 °C and 0.10 °C increase in CT_max_ per 1 °C increase in acclimation temperature respectively. Previously, Terblanche and Chown (2006) found a limited, or non-existent, response of CT_max_ to temperature acclimation at 21, 25, and 29 °C in *G. pallidipes*. Therefore, our findings indicate that thermal tolerance plasticity is not fully constrained among closely related tsetse species. In general, CT_max_ of ectotherms respond relatively weakly to thermal acclimation (Gunderson and Stillman, 2015). Insects on average show a 0.09 °C rise in CT_max_ per 1 °C acclimation temperature (Weaving et al., 2022). Studies have found that CT_max_ is constrained within a narrower range than CT_min_, which may reflect hard physiological limits at high temperature (Sandblom et al., 2016). For example, both CT_max_ and CT_min_ decline with increasing latitude, but CT_max_ is an order of magnitude less responsive (Sunday et al., 2011). Evolutionary and plastic constraints to CT_max_ are worrying for insects and other ectotherms given ever increasing mean and maximum temperatures due to climate change.

Differences in CT_max_ plasticity could not be explained by body size or sex in tsetse. However, our meta-analysis of Diptera indicated that males were more plastic than females by 0.03 °C per 1 °C increase in acclimation temperature. This is in opposition to a recent meta-analysis on acclimation in ectotherms which found that females are more plastic than males in wild-caught populations (Pottier et al., 2021). Male-associated behaviours, such as large home ranges and increased risk taking (Tarka et al., 2018; Todd and Nowakowski, 2021), may be more sexually divergent in Diptera than in other ectothermic species. Broad-scale analyses on many phylogenetic groups may obscure trends if there are opposing selection pressures between groups.

We found that basal CT_max_ differed among species, and differences were related to intra-species relationships with body size and sex. Larger flies generally had greater CT_max_ within *G. pallidipes, G. m. morsitans* and *G. brevipalpis*. Correspondingly, differences between male and female tsetse mirrored trends for body size, indicating that differences are likely related to female tsetse being larger than male tsetse, a rule common across insects (Honěk, 1993). These patterns may have been due to larger individuals having more energy reserves and a slower metabolic rate to size ratio, or alternatively, greater thermal inertia of larger individuals may have slowed the rate at which their body temperature increased (Brown et al., 2004; Stevenson, 1985). Indeed, field studies show that in hotter months, small bodied tsetse are selectively eliminated which could be owing to their lower CT_max_ (Bursell and Glasgow, 1960; Jackson, 1948). In addition, insects raised under high temperature tend to be smaller adults (Kingsolver and Huey, 2008), which is also true for tsetse (Weaving et al., 2023). Therefore, high temperatures may have two effects − first, development temperature may result in the emergence of small-bodied flies, and, second, these flies may have subsequently lower basal CT_max_ and therefore have higher mortality during hot periods.

We note, however, that body size trends did not apply among species: *G. brevipalpis* is the largest species but had the lowest basal CT_max_. Species differences may be due to different distributions and, therefore, thermal history. Source locations of the five tsetse species considered here are presented in Supplementary Table 1 and range from approximately zero to 20° latitude. We did not find any clear evidence linking latitude to CT_max_ and, given that only five of the total 31 tsetse species and subspecies were measured, we caveat that any broader generalisations from our findings are speculative. *Glossina palpalis gambiensis* and *G. brevipalpis* had the lowest basal CT_max_ but showed the largest acclimation response (Fig. 2). Individuals with lower basal tolerance may exhibit greater plasticity, known as the tolerance-plasticity trade-off hypothesis (van Heerwaarden and Kellermann, 2020). The implications of this hypothesis are that species with the highest basal tolerances may be more vulnerable to temperature rises due to their lack of plasticity. However, findings in support of this hypothesis (e.g. Comte and Olden, 2017; Faulkner et al., 2014; Vinagre et al., 2018) have recently come under scrutiny due to statistical issues of collinearity and regression to the mean, and if true, these findings may be artifacts of experimental design and statistical analysis (Gunderson, 2023; Gunderson and Revell, 2022; van Heerwaarden and Kellermann, 2020). Undoubtedly, we would recommend testing more tsetse species before coming to any conclusions.

### 4.1 Conclusions

Overall, we show intra- and inter-specific differences in CT_max_ and its plasticity across tsetse species. In general, plasticity of CT_max_ was weak, in agreement with studies which show a reduction in the range tsetse are likely to inhabit due to climate change (Are and Hargrove, 2020; Longbottom et al., 2020). Moreover, we argue that warming temperatures will result in smaller body sizes, which is associated with reduced CT_max_, and thus will further constrain capacity to cope with climate change across multiple tsetse species. Our study highlights that broad patterns are not always reflected within closely related species and or even within species, therefore detailed experimental studies are needed to capture the capacity of insects to cope with rapidly warming temperatures.

## Supplementary Material

Supplementary data to this article can be found online at https://doi.org/10.1016/j.jtherbio.2023.103745.

Supplementary data

## Figures and Tables

**Table 1:** Reaction norm slope pairwise comparisons for five species of tsetse (*Glossina* spp.). Slopes indicate the plasticity of each species at the population level as means of each acclimation group per species. Mean differences between slopes are given ± Standard Error (SE). The Tukey method was used for P-value adjustment, comparing a family of five estimates. Species are presented in order of most to least plastic.

Species	Contrast	Mean difference ±SE	DF	t- statistic	P value
*G. p. gambiensis*	*G. brevipalpis*	0.05 ± 0.15	378	0.34	>0.99
	*G. pallidipes*	**0.56 ± 0.15**	**378**	**3.77**	**0.002**
	*G. f. fuscipes*	**0.48 ± 0.15**	**378**	**3.24**	**0.01**
	*G. m. morsitans*	**0.42 ± 0.15**	**378**	**2.80**	**0.04**
*G. brevipalpis*	*G. pallidipes*	**0.50 ± 0.15**	**378**	**3.41**	**0.006**
	*G. f. fuscipes*	**0.43 ± 0.15**	**378**	**2.89**	**0.03**
	*G. m. morsitans*	0.37 ± 0.15	378	2.45	0.10
*G. m. morsitans*	*G. pallidipes*	0.14 ± 0.15	378	0.92	0.89
	*G. f. fuscipes*	0.063 ± 0.15	378	0.42	0.99
*G. f. fuscipes*	*G. pallidipes*	0.074 ± 0.15	378	0.50	0.99

**Table 2:** Main intercept and univariate multi-level meta-analytic, random effects models for (CT_max_) critical thermal maximum. The main model tests whether ARR (Acclimation Response Ratio) is significantly different from zero, and univariate models are regressions or compare differences between moderator groups. Results for intercept models are displayed. Results are highlighted in bold where 95% CIs do not overlap between groups or where regressions are significant for continuous variables. CI.lb: lower bound of the 95% confidence interval; CI.ub: upper bound of the 95% confidence interval. I^2^ is the proportion of heterogeneity explained by each of the random effects. R^2^ marg.: R^2^ marginal, the variance explained only by moderators. R^2^ cond.: R^2^ conditional, the variance explained by moderators and random effects.

Model	Group	Est.	t	CI.lb	CI.ub	AICc	k	I^2^				R^2^	
								study	phylogeny	species	row	marg.	cond.
Main ∼mass	**Intercept**	**0.048**	**4.07**	**0.024**	**0.072**	−691.9	488	1.47	<0.001	16.2	79.6	−	0.18
	**Intercept**	**0.046**	**3.17**	**0.016**	**0.077**	−615.2	453	1.41	<0.001	18.4	77.7	<0.001	0.20
		0.0002	0.16	−0.002	0.002								
∼sex	**Intercept (female)**	**0.038**	**2.96**	**0.011**	**0.064**	−688.3	488	<0.001	<0.001	18.0	79.2	0.013	0.20
	**Male**	**0.026**	**2.20**	**0.003**	**0.048**								
	Both	−0.002	−0.08	−0.061	0.056								
∼family	Intercept (Glossin.)	0.048	0.71	−0.093	0.188	−679.4	488	1.78	23.0	10.4	62.6	0.009	0.37
	Culic.	0.018	0.19	−0.180	0.216								
	Drosophil.	−0.014	−0.20	−0.166	0.137								
	Ceratopog.	0.057	0.47	−0.197	0.310								
	Tephrit.	0.005	0.09	−0.195	0.185								

## References

[R1] Addo-Bediako A, Chown SL, Gaston KJ (2000). Thermal tolerance, climatic variability and latitude. Proc R Soc B Biol Sci.

[R2] Allen JL, Clusella-Trullas S, Chown SL (2012). The effects of acclimation and rates of temperature change on critical thermal limits in *Tenebrio molitor* (Tenebrionidae) and *Cyrtobagous salviniae* (Curculionidae). J Insect Physiol.

[R3] Angilletta MJ (2009). Thermal Adaptation: a Theoretical and Empirical Synthesis.

[R4] Are EB, Hargrove JW (2020). Extinction probabilities as a function of temperature for populations of tsetse (*Glossina* spp). PLoS Neglected Trop Dis.

[R5] Bates D, Machler M, Bolker B, Walker S (2015). Fitting linear mixed-effects models using lme4. J Stat Software.

[R6] Baudier K, O’Donnell S (2018). Complex body size differences in thermal tolerance among army ant workers (*Eciton burchellii parvispinum*). J Therm Biol.

[R7] Belliard SA, De La Vega GJ, Schilman PE (2019). Thermal tolerance plasticity in chagas disease vectors Rhodnius prolixus (Hemiptera: reduviidae) and *Triatoma infestans*. J Med Entomol.

[R8] Bergmann C (1847). Ueber die Verhältnisse der Wärmeökonomie der Thiere zu ihrer Grösse. Gottingerstudien.

[R9] Bradburn MJ, Clark TG, Love SB, Altman DG (2003). Survival Analysis Part II: multivariate data analysis-an introduction to concepts and methods. Br J Cancer.

[R10] Brown J, Gillooly JF, Allen AP, Savage VM, West G (2004). Toward a metabolic theory of ecology. Ecology.

[R11] Bulté G, Blouin-Demers G (2010). Implications of extreme sexual size dimorphism for thermoregulation in a freshwater turtle. Oecologia.

[R12] Burger R, Lynch M (1995). Evolution and extinction in a changing environment: a quantitative-genetic analysis. Evolution.

[R13] Bursell E, Glasgow JP (1960). Further observations on lake-side and riverine communities of *Glossina palpalis fuscipes* Newstead. Bull Entomol Res.

[R14] Buxton PA (1955). The Natural History of Tsetse Flies. An Account of the Biology of the Genus *Glossina* (Diptera), London School of Hygiene and Tropical Medicine.

[R15] Chapelle G, Peck LS (1999). Polar gigantism dictated by oxygen availability. Nature.

[R16] Chown SL, Sørensen JG, Terblanche JS (2011). Water loss in insects: an environmental change perspective. J Insect Physiol.

[R17] Christidis N, Jones GS, Stott PA (2015). Dramatically increasing chance of extremely hot summers since the 2003 European heatwave. Nat Clim Change.

[R18] Clusella-Trullas S, Chown SL (2014). Lizard thermal trait variation at multiple scales: a review. J Comp Physiol B Biochem Syst Environ Physiol.

[R19] Comte L, Olden JD (2017). Evolutionary and environmental determinants of freshwater fish thermal tolerance and plasticity. Global Change Biol.

[R20] Cossins AR, Bowler K (1987). Temperature Biology of Animals.

[R21] Couper LI, Farner JE, Caldwell JM, Childs ML, Harris MJ, Kirk DG, Nova N, Shocket M, Skinner EB, Uricchio LH, Exposito-Alonso M (2021). How will mosquitoes adapt to climate warming?. Elife.

[R22] Donelson JM, Salinas S, Munday PL, Shama LNS (2018). Transgenerational plasticity and climate change experiments: where do we go from here?. Global Change Biol.

[R23] Faulkner KT, Clusella-Trullas S, Peck LS, Chown SL (2014). Lack of coherence in the warming responses of marine crustaceans. Funct Ecol.

[R24] García-Robledo C, Kuprewicz EK, Staines CL, Erwin TL, Kress WJ (2016). Limited tolerance by insects to high temperatures across tropical elevational gradients and the implications of global warming for extinction. Proc Natl Acad Sci USA.

[R25] Gardner JL, Peters A, Kearney MR, Joseph L, Heinsohn R (2011). Declining body size: a third universal response to warming? Trends Ecol. Evol.

[R26] Garnier S, Ross N, Rudis R, Camargo PA, Sciaini M, Scherer C (2023). viridis(Lite) - Colorblind-Friendly Color Maps for R.

[R27] Gunderson AR (2023). Trade-offs between baseline thermal tolerance and thermal tolerance plasticity are much less common than it appears. Global Change Biol.

[R28] Gunderson AR, Revell LJ (2022). Testing for genetic assimilation with phylogenetic comparative analysis: conceptual, methodological, and statistical considerations. Evolution.

[R29] Gunderson AR, Stillman JH (2015). Plasticity in thermal tolerance has limited potential to buffer ectotherms from global warming. Proc Biol Sci.

[R30] Hargrove JW (2004). Tsetse population dynamics. The Trypanosomiases CABI.

[R31] Hay SI, Guerra CA, Tatem AJ, Noor AM, Snow RW (2004). The global distribution and population at risk of malaria: past, present, and future. Lancet Infect Dis.

[R32] Honěk A (1993). Intraspecific variation in body size and fecundity in insects: a general relationship. Oikos.

[R33] Jackson CH (1948). The analysis of a tsetse-fly population. III. Ann Eugen.

[R34] Jackson CHN (1946). An artificially isolated generation of tsetse flies (Diptera. Bull Entomol Res.

[R35] Kellermann V, Overgaard J, Hoffmann AA, Fljøgaard C, Svenning JC, Loeschcke V (2012). Upper thermal limits of *Drosophila* are linked to species distributions and strongly constrained phylogenetically. Proc Natl Acad Sci USA.

[R36] Kingsolver JG, Huey RB (2008). Size, temperature, and fitness: three rules. Evol Ecol Res.

[R37] Leak SGA (1998). Tsetse Biology and Ecology: Their Role in the Epidemiology and Control of Trypanosomosis.

[R38] Lenth RV (2016). Least-squares means: the R package lsmeans. J Stat Software.

[R39] Longbottom J, Caminade C, Gibson HS, Weiss DJ, Torr S, Lord JS (2020). Modelling the impact of climate change on the distribution and abundance of tsetse in Northern Zimbabwe. Parasites Vectors.

[R40] Lord JS, Hargrove JW, Torr SJ, Vale GA (2018). Climate change and African trypanosomiasis vector populations in Zimbabwe’s Zambezi Valley: a mathematical modelling study. PLoS Med.

[R41] MacMillan HA, Nørgård M, MacLean HJ, Overgaard J, Williams CJA (2017). A critical test of *Drosophila anaesthetics*: isoflurane and sevoflurane are benign alternatives to cold and CO2. J Insect Physiol.

[R42] Meehl GA, Tebaldi C (2004). More intense, more frequent, and longer lasting heat waves in the 21st century. Science.

[R43] Michonneau F, Brown J, Winter D (2016). rotl: an R package to interact with the Open Tree of Life data. Methods Ecol Evol.

[R44] Nakagawa S, Lagisz M, Jennions MD, Koricheva J, Noble DWA, Parker TH, Sánchez-Tójar A, Yang Y, O’Dea R (2022). Methods for testing publication bias in ecological and evolutionary meta-analyses. Methods Ecol Evol.

[R45] Nakagawa S, Lagisz M, O’Dea RE, Rutkowska J, Yang Y, Noble DWA, Senior AM (2021). The orchard plot: cultivating a forest plot for use in ecology, evolution, and beyond. Res Synth Methods.

[R46] Oliveira BF, Yogo WIG, Hahn DA, Yongxing J, Scheffers BR (2021). Community-wide seasonal shifts in thermal tolerances of mosquitoes. Ecology.

[R47] Opiyo E, Parker A, Mohammed A FAO/IAEA Standard Operating Procedures for Mass-Rearing Tsetse Flies. https://www.iaea.org/resources/manual/standard-operating-procedures-for-mass-rearing-tsetse-flies.

[R48] Overgaard J, Kearney MR, Hoffmann AA (2014). Sensitivity to thermal extremes in Australian *Drosophila* implies similar impacts of climate change on the distribution of widespread and tropical species. Global Change Biol.

[R49] Pagabeleguem S, Ravel S, Dicko AH, Vreysen MJB, Parker A, Takac P, Huber K, Sidibé I, Gimonneau G, Bouyer J (2016). Influence of temperature and relative humidity on survival and fecundity of three tsetse strains. Parasites Vectors.

[R50] Paradis E, Schliep K (2019). Ape 5.0: an environment for modern phylogenetics and evolutionary analyses in R. Bioinformatics.

[R51] Peck LS, Clark MS, Morley SA, Massey A, Rossetti H (2009). Animal temperature limits and ecological relevance: effects of size, activity and rates of change. Funct Ecol.

[R52] Peralta-Maraver I, Rezende EL (2021). Heat tolerance in ectotherms scales predictably with body size. Nat Clim Change.

[R53] Perkins SE, Alexander LV, Nairn JR (2012). Increasing frequency, intensity and duration of observed global heatwaves and warm spells. Geophys Res Lett.

[R54] Pincebourde S, Dillon ME, Woods HA (2021). Body size determines the thermal coupling between insects and plant surfaces. Funct Ecol.

[R55] Pörtner HO (2010). Oxygen- and capacity-limitation of thermal tolerance: a matrix for integrating climate-related stressor effects in marine ecosystems. J Exp Biol.

[R56] Pottier P, Burke S, Drobniak SM, Lagisz M, Nakagawa S (2021). Sexual (in) equality? A meta-analysis of sex differences in thermal acclimation capacity across ectotherms. Funct Ecol.

[R57] R Core Team (2021). R: A Language and Environment for Statistical Computing.

[R58] Recsetar MS, Zeigler MP, Ward DL, Bonar SA, Caldwell CA (2012). Relationship between fish size and upper thermal tolerance. Trans Am Fish Soc.

[R59] Rogers D, Randolph S (1993). Simple multi-variate analysis of vector distributions. Parasitol Today.

[R60] Rohatgi A (2010). WebPlotDigitizer - Extract Data from Plots, Images, and Maps. Arohatgi.

[R61] Rohr JR, Civitello DJ, Cohen JM, Roznik EA, Sinervo B, Dell AI (2018). The complex drivers of thermal acclimation and breadth in ectotherms. Ecol Lett.

[R62] Sandblom E, Clark TD, Gräns A, Ekström A, Brijs J, Sundström LF, Odelström A, Adill A, Aho T, Jutfelt F (2016). Physiological constraints to climate warming in fish follow principles of plastic floors and concrete ceilings. Nat Commun.

[R63] Seebacher F, White CR, Franklin CE (2015). Physiological plasticity increases resilience of ectothermic animals to climate change. Nat Clim Change.

[R64] Sheridan JA, Bickford D (2011). Shrinking body size as an ecological response to climate change. Nat Clim Change.

[R65] Simarro PP, Cecchi G, Franco JR, Paone M, Diarra A, Ruiz-Postigo JA, Fèvre EM, Mattioli RC, Jannin JG (2012). Estimating and mapping the population at risk of sleeping sickness. PLoS Neglected Trop Dis.

[R66] Smith JJ, Hasiotis ST, Kraus MJ, Woody DT (2009). Transient dwarfism of soil fauna during the paleocene-eocene thermal maximum. Proc Natl Acad Sci USA.

[R67] Stevenson RD (1985). Body size and limits to the daily range of body temperature in terrestrial ectotherms. Am Nat.

[R68] Stillwell RC, Blanckenhorn WU, Teder T, Davidowitz G, Fox CW (2010). Sex differences in phenotypic plasticity affect variation in sexual size dimorphism in insects: from physiology to evolution. Annu Rev Entomol.

[R69] Sunday JM, Bates AE, Dulvy NK (2011). Global analysis of thermal tolerance and latitude in ectotherms. Proc R Soc B Biol Sci.

[R70] Tarka M, Guenther A, Niemelä PT, Nakagawa S, Noble DWA (2018). Sex differences in life history, behavior, and physiology along a slow-fast continuum: a meta-analysis. Behav Ecol Sociobiol.

[R71] Terblanche JS, Chown SL (2006). The relative contributions of developmental plasticity and adult acclimation to physiological variation in the tsetse fly, *Glossina pallidipes* (Diptera, Glossinidae). J Exp Biol.

[R72] Todd BD, Nowakowski AJ (2021). Ectothermy and the macroecology of home range scaling in snakes. Global Ecol Biogeogr.

[R73] van Heerwaarden B, Kellermann V (2020). Does plasticity trade off with basal heat tolerance? Trends Ecol. Evol.

[R74] Verberk WCEP, Leuven RSEW, van der Velde G, Gabel F (2018). Thermal limits in native and alien freshwater peracarid Crustacea: the role of habitat use and oxygen limitation. Funct Ecol.

[R75] Viechtbauer W (2010). Conducting meta-analyses in R with the metafor. J Stat Software.

[R76] Vinagre C, Mendonça V, Cereja R, Abreu-Afonso F, Dias M, Mizrahi D, Flores AAV (2018). Ecological traps in shallow coastal waters-Potential effect of heat-waves in tropical and temperate organisms. PLoS One.

[R77] von May R, Catenazzi A, Santa-Cruz R, Gutierrez AS, Moritz C, Rabosky DL (2019). Thermal physiological traits in tropical lowland amphibians: vulnerability to climate warming and cooling. PLoS One.

[R78] Vorhees AS, Bradley TJ (2012). Differences in critical thermal maxima and mortality across life stages of the mealworm beetle *Tenebrio molitor*. J Exp Biol.

[R79] Weaving H, Lord JS, Haines L, English S (2023). No evidence for direct thermal carryover effects on starvation tolerance in the obligate blood-feeder, Glossina morsitans morsitans. Ecol Evol.

[R80] Weaving H, Terblanche JS, Pottier P, English S (2022). Meta-analysis reveals weak but pervasive plasticity in insect thermal limits. Nat Commun.

[R81] Wickham H (2016). ggplot2: Elegant Graphics for Data Analysis.

[R82] Zhang Y, Kieffer JD (2014). Critical thermal maximum (CTmax) and hematology of shortnose sturgeons (*Acipenser brevirostrum*) acclimated to three temperatures. Can J Zool.

