# Supplementary material for: How plastic are upper thermal limits? A comparative study in tsetse (family: Glossinidae) and wider Diptera
Source: J Therm Biol. Author manuscript; Available in PMC 2025 Jun 7. (PMC7617741; doi:10.1016/j.jtherbio.2023.103745)
Supplement: Supplementary data [file EMS204586-supplement-Supplementary_data.zip › 1-s2.0-S0306456523002863-mmc1.docx]

**Supplementary material**


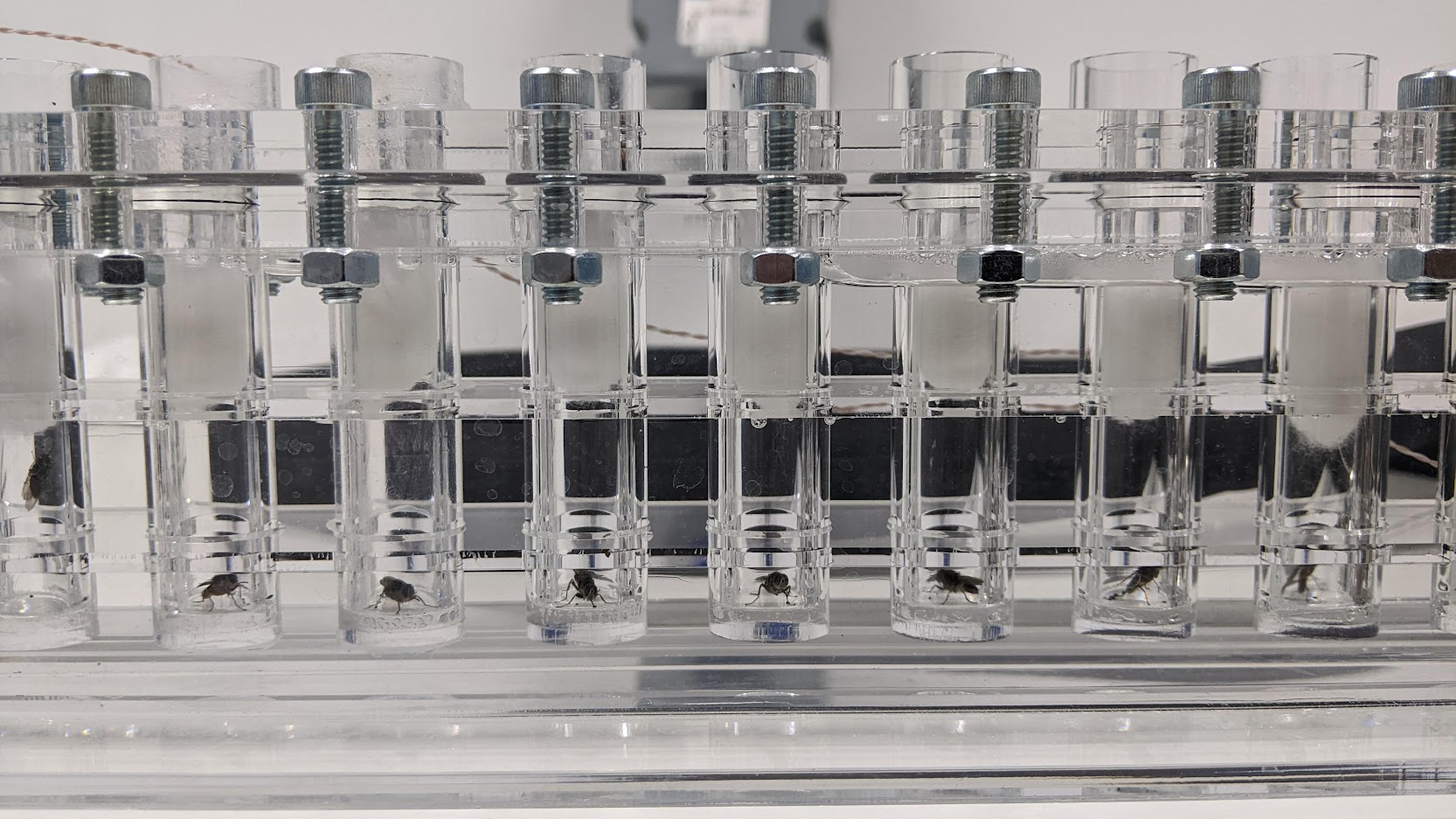


**Supplementary Figure 1** Organ pipe set up for CT_max_ experiments. Organ pipes are connected by tubing to Grant LTC4 circulating water baths. Temperature of the inside of the pipes was taken by T type thermocouple. Seen here, *G. pallidipes.*

**Supplementary Table 1** Mean ± SE temperature and relative humidity of acclimation treatments for each species recorded by iButton data logger. Origin is the location where flies were originally collected for colony establishment by IAEA.

| Species | Sub-genera | Origin | Treatment | Temperature ± SD | Humidity ± SD |
| --- | --- | --- | --- | --- | --- |
| *G. brevipalpis* | Fusca | Kibwezi Forest Kenya | 25 | 24.9 ± 0.3 | 78.8 ± 0.6 |
|  |  |  | 30 | 30.6 ± 1.1 | 81.6 ± 4.8 |
| *G. f. fuscipes* | Palpalis | Central African Republic | 25 | 24.9 ± 0.3 | 78.5 ± 0.7 |
|  |  |  | 30 | 30.8 ± 0.8 | 78.6 ± 3.7 |
| *G. p. gambiensis* | Palpalis | Burkina Faso, Pout Senegal | 25 | 24.9 ± 0.3 | 78.8 ± 0.6 |
|  |  |  | 30 | 30.5 ± 0.6 | 79.9 ± 5.9 |
| *G. m. morsitans* | Morsitans | Zimbabwe | 25 | 24.9 ± 0.3 | 78.8 ± 0.6 |
|  |  |  | 30 | 30.4 ± 1.0 | 81.6 ± 5.0 |
| *G. pallidipes* | Morsitans | Tororo Uganda | 25 | 25.2 ± 0.1 | 76.3 ± 1.4 |
|  |  |  | 30 | 30.7 ± 0.1 | 78.0 ± 2.36 |

**Supplementary Table 2** Mortality rates of *Glossina* spp. one week after treatment with sevoflurane. Sevoflurane (350 μl) was applied to cotton wool for one cage of approximately 25 flies in an enclosed plastic container (20 x 25 x 10 cm) for 10 minutes.

| Species | Sevoflurane treatment | No. alive | No. dead | Proportion alive | N cages |
| --- | --- | --- | --- | --- | --- |
| *G. brevipalpis* | Treated | 178 | 2 | 0.99 | 9 |
|  | Untreated | 112 | 3 | 0.97 | 6 |
| *G. pallidipes* | Treated | 158 | 12 | 0.93 | 8 |
|  | Untreated | 56 | 3 | 0.95 | 3 |
| *G. f. fuscipes* | Treated | 146 | 15 | 0.91 | 8 |
|  | Untreated | 125 | 8 | 0.94 | 7 |
| *G. m. morsitans* | Treated | 127 | 15 | 0.89 | 7 |
|  | Untreated | 121 | 11 | 0.92 | 7 |
| *G. p. gambiensis* | Treated | 63 | 0 | 1.00 | 9 |
|  | Untreated | 54 | 0 | 1.00 | 4 |

**Supplementary Table 3** Critical Thermal Maximum (CT_max_) mean ± Standard Error (SE) of tsetse flies (*Glossina* spp.) acclimated to either 25°C (basal) or 30°C for five consecutive days. CT_max_ was measured by ramping assay beginning at 25°C and ramping at 0.1°C/min.

| Species | Adult acclimation temperature | CT_max_ (°C) | SE | N |
| --- | --- | --- | --- | --- |
| *G. brevipalpis* | 25 | 41.3 | 0.11 | 40 |
|  | 30 | 41.8 | 0.10 | 40 |
| *G. pallidipes* | 25 | 42.9 | 0.04 | 40 |
|  | 30 | 42.9 | 0.05 | 40 |
| *G. f. fuscipes* | 25 | 43.1 | 0.64 | 39 |
|  | 30 | 43.2 | 0.74 | 39 |
| *G. m. morsitans* | 25 | 42.6 | 0.08 | 38 |
|  | 30 | 42.8 | 0.09 | 40 |
| *G. p. gambiensis* | 25 | 42.6 | 0.07 | 40 |
|  | 30 | 43.2 | 0.06 | 40 |

**Supplementary Table 4** Single-species linear models investigating the effect of acclimation and sex on Critical Thermal Maximum (CT_max_) in tsetse (*Glossina* spp.). Flies were either acclimated at 25 or 30 for five days preceding the assay. Mean differences between groups and slopes are given ± SE.

| Model | Variable | Mean difference ± SE | t-statistic | P-value |  | F-statistic | DF | P-value | R^2^ |
| --- | --- | --- | --- | --- | --- | --- | --- | --- | --- |
|  | Parameters | | | |  | Model | | | |
| *G. brevipalpis* | **Intercept (25°C)** | **41.47 ± 0.12** | **332.0** | **<0.0001** |  | **8.95** | **2, 76** | **<0.001** | **0.19** |
|  | **Treatment (30°C)** | **0.52 ± 0.14** | **3.61** | **<0.001** |  |  |  |  |  |
|  | **Sex (female - male)** | **-0.33 ± 0.14** | **-2.26** | **0.03** |  |  |  |  |  |
| *G. pallidipes* | **Intercept (25°C)** | **43.03 ± 0.05** | **827.9** | **<0.0001** |  | **9.76** | **2, 77** | **<0.001** | **0.20** |
|  | Treatment (30°C) | 0.04 ± 0.06 | 0.63 | 0.53 |  |  |  |  |  |
|  | **Sex (female - male)** | **-0.26 ± 0.06** | **-4.37** | **<0.0001** |  |  |  |  |  |
| *G. f. fuscipes* | **Intercept (25°C)** | **43.02 ± 0.10** | **451.7** | **<0.0001** |  | 1.77 | 2, 74 | 0.16 | 0.07 |
|  | **Treatment (30°C)** | **0.30 ± 0.14** | **2.21** | **0.03** |  |  |  |  |  |
|  | Sex (female - male) | 0.22 ± 0.14 | 1.59 | 0.12 |  |  |  |  |  |
|  | Treatment x sex | -0.38 ± 0.19 | -1.98 | 0.05 |  |  |  |  |  |
| *G. m. morsitans* | **Intercept (25°C)** | **42.96 ± 0.08** | **516.3** | **<0.0001** |  | **25.44** | **2, 74** | **<0.0001** | **0.41** |
|  | Treatment (30°C) | 0.17 ± 0.10 | 1.79 | 0.08 |  |  |  |  |  |
|  | **Sex (female - male)** | **-0.68 ± 0.10** | **-6.97** | **<0.0001** |  |  |  |  |  |
| *G. p. gambiensis* | **Intercept (25°C)** | **42.62 ± 0.08** | **505.9** | **<0.0001** |  | **19.61** | **2, 77** | **<0.0001** | **0.34** |
|  | **Treatment (30°C)** | **0.60 ± 0.10** | **6.17** | **<0.0001** |  |  |  |  |  |
|  | Sex (female - male) | -0.11 ± 0.10 | -1.08 | 0.28 |  |  |  |  |  |


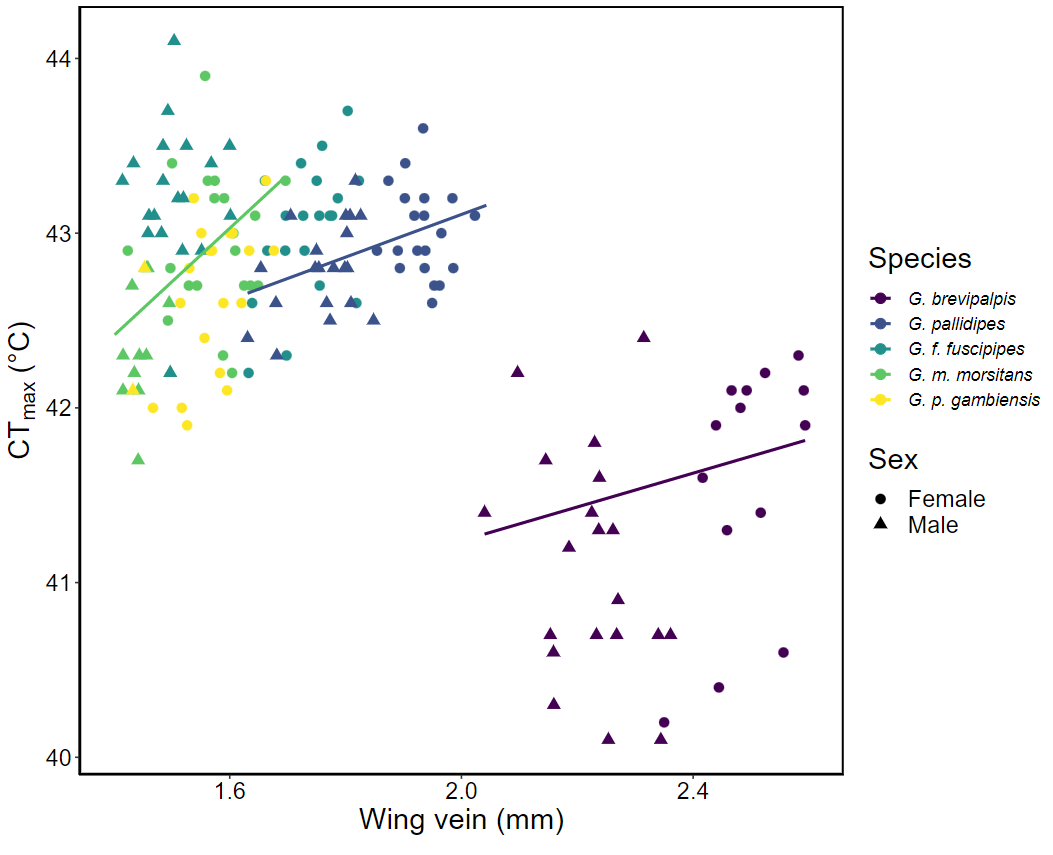


**Supplementary Figure 2** Relationship between basal CT_max_ and wing vein size (mm). *Glossina* spp. are distinguished by different colours. Lines represent significant linear regressions within species groups. The linear regression for *G. brevipalpis* was near to significant (p = 0.08) so has been included. Circles resemble female flies and triangles resemble males. Species are given in mean size order from largest to smallest by dry mass.


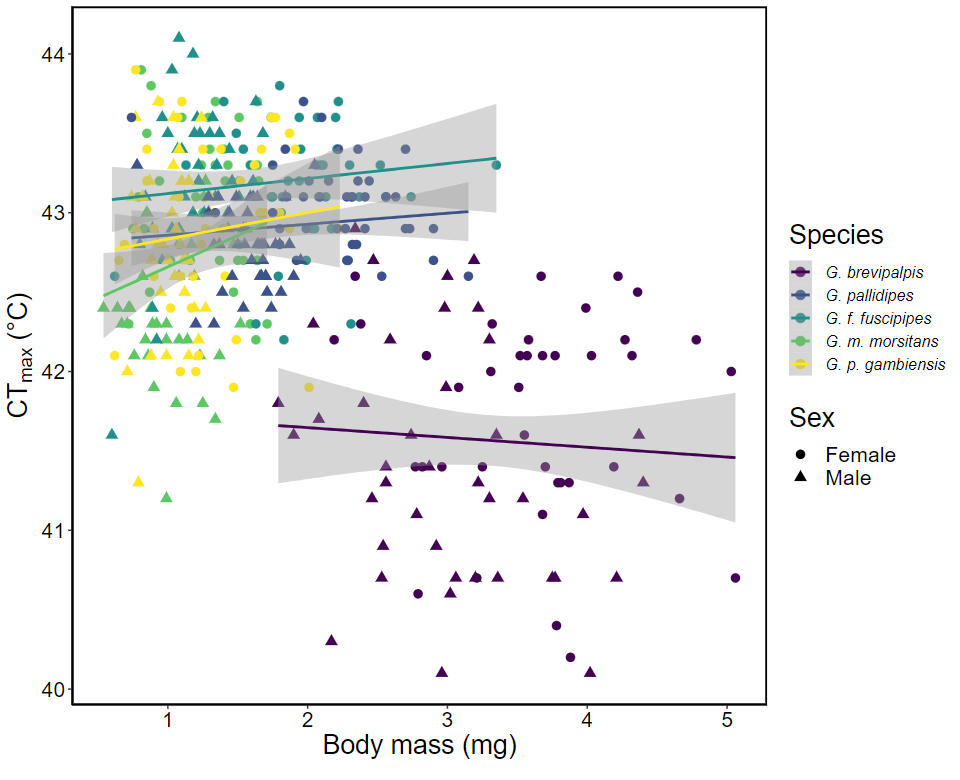


**Supplementary Figure 3** Relationship between CT_max_ and body mass (mg). *Glossina* spp. are distinguished by different colours. Lines represent linear regressions within species groups. Circles resemble female flies and triangles resemble males. CT_max_ is represented for individuals acclimated at both 25°C and 30°C. N ~ 40 per treatment/sex/species.


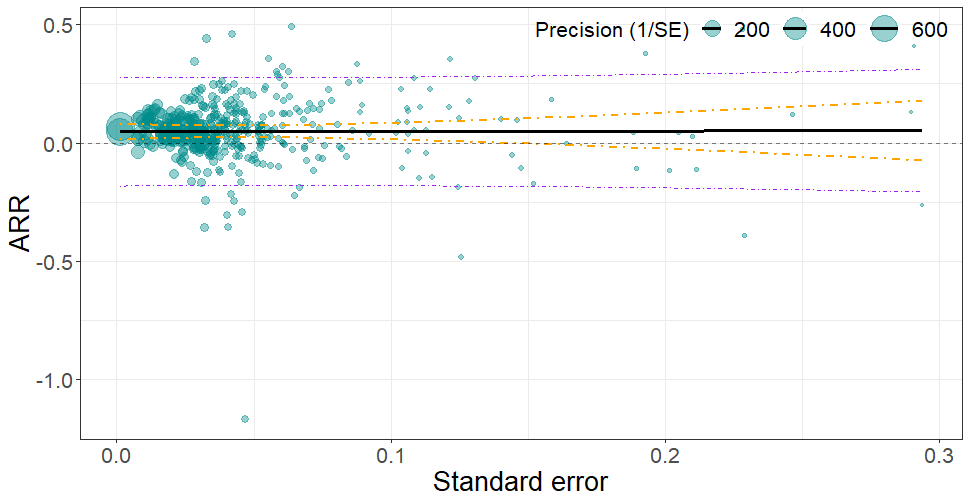


**Supplementary Figure 4** Relationship between Critical Thermal Maximum ARR (Acclimation Response Ratio) and standard error for Egger’s regression test. A positive relationship shows positive publication bias. 95% confidence intervals are depicted by orange dotted lines, prediction intervals are purple dotted lines. The precision of the study (1/SE) is proportional to the size of each data point.
